# Oncosuppressors and Oncogenes: Role in Haemangioma Genesis and Potential for Therapeutic Targeting

**DOI:** 10.3390/ijms19041192

**Published:** 2018-04-13

**Authors:** Peace Mabeta

**Affiliations:** Department of Physiology, Faculty of Health Sciences, University of Pretoria, 9 Botshelo Road, Pretoria 0007, South Africa; peace.mabeta@up.ac.za; Tel.: +27-012-319-29-07; Fax: +27-012-321-16-79

**Keywords:** angiogenesis, hemangioma, oncogene, oncosuppressor, vascular endothelial growth factor

## Abstract

Genetic lesions in proto-oncogenes result in the perturbation of angiogenesis, the formation of neovessels from a pre-existing microvasculature. Similarly, the subversion of tumor suppressor genes promotes tumor vascularization. Excessive neovessel formation is associated with various neoplasms such as infantile hemangiomas (IH). Hemangiomas are the most common tumors in pediatric patients and at present have no definitive treatment. The pathogenesis of IH is not well understood; however, both vasculogenesis and angiogenesis are associated with hemangioma genesis. A number of factors that modulate angiogenesis and vasculogenesis have been shown to be dysregulated in IH. Several of the oncogenes and tumor suppressors linked to the promotion of angiogenesis are also altered in infantile hemangioma. In this review, the roles of oncogenes and tumor suppressor genes during neovascularization and hemangioma genesis are explored. In addition, the potential for targeting these genes in IH therapy is discussed.

## 1. Introduction

Infantile hemangioma (IH) is deemed the most common tumor of infancy. It is estimated that IH develops in 10% of infants and that the frequency of hemangioma development increases to 22.9% for premature infants with a birth weight below 1 kg [1,2]. The tumors have a life-cycle of three phases. The first phase, known as the proliferative phase, is characterized by an increase in the proliferation of endothelial cells (ECs) [1]. The second or involuting phase is characterized by a decrease in EC turnover. During the third or involuted phase, there is complete resolution of the tumor with replacement of vascular tissue by fibro-fatty tissue [1]. Most IH involute spontaneously; however, a subset of the lesions may be disfiguring, while some may impair function or be potentially life-threatening [3]. Such lesions require treatment. For many years, steroids have been the mainstay treatment for problematic IH [3]. At present, β-blockers represent the first-line treatment for IH [4]. Current treatment modalities are not without limitations, and locally-administered β-blockers are mainly effective in the treatment of superficial lesions [4,5]. Thus, understanding the precise pathways underlying hemangioma genesis will be useful for devising targeted and more effective therapies.

Several theories have been proposed to explain the pathogenesis of IH. Clonal expansion of a stem cell carrying a somatic mutation in a critical gene, tissue hypoxia, as well as an abnormal hormonal milieu, such as an increase in estrogen levels, have been postulated as potential stimuli for hemangioma genesis [6,7]. While IH may develop due to one or several of the postulated defects, the common underlying feature in the pathogenesis of IH is excessive neovascularization [3,8].

There is a strong correlation between the subversion of tumor suppressor genes and angiogenesis, a process that is associated with proliferating IH [9,10]. In addition, oncogenic modifications promote tumor angiogenesis [11]. Many of the altered tumor suppressors and oncogenes promote tumor neovascularization in part by affecting vascular endothelial growth factor (VEGF) signaling [12]. This review explores the genetic alterations associated with the promotion of the neovascularization in IH, as well as possible therapeutic approaches that can be employed to exploit these genetic perturbations. 

## 2. Vascular Endothelial Growth Factor

The vascular endothelial growth factor (VEGF) family is a group of secreted glycoproteins that consists of VEGF-A, -B, -C, -D and placental growth factor (PlGF) [13,14]. The most characterized angiogenic growth factor in this family, VEGF-A (referred to in this review as VEGF), is a required for vasculogenesis, the development of blood vessels from primordial stem cells [14]. Vascular endothelial growth factor is also an important mediator of embryonic and postnatal angiogenesis [14,15].

### 2.1. Vascular Endothelial Growth Factor Signaling in Angiogenesis

The VEGF family of ligands binds to receptor tyrosine kinases (RTKs), and the availability of the VEGF ligands regulates the activity of the vascular endothelial growth factor receptors (VEGFRs) [14]. Vascular endothelial growth factor-B (VEGF-B) and placental growth factor (PlGF) bind to vascular endothelial growth factor receptor-1 (VEGFR-1) or *Fms*-like tyrosine kinase 1 (FLT-1). The ligands vascular endothelial growth factor C (VEGF-C) and vascular endothelial growth factor D (VEGF-D) bind to VEGFR-3 to promote lymphangiogenesis, the formation of lymphatic vessels from pre-existing lymphatic vessels. On the other hand, the principal receptors for VEGF are VEGFR-1 and VEGFR-2/fetal liver kinase 1 (Flk-1) or kinase insert domain receptor (KDR) [16]. These receptors are mainly expressed by endothelial cells, although some tumor cells do express VEGF receptors [15]. 

In endothelial cells, VEGF modulates mitogenic signals by activating both VEGFR-1 and -2 [13,14]. Vascular endothelial growth factor receptor-2 has less affinity for VEGF when compared with VEGFR-1; however, VEGFR-2 presents greater signaling activity [13,15,16]. As such, the mitogenic action of VEGF in endothelial cells is mediated mainly by VEGFR-2 [14,15]. Additionally, VEGFR-2 mediates cell leakage and vascular permeability in response to VEGF, whereas VEGFR-1 has a weak or undetectable response [13]. Furthermore, the signaling pathways elicited by VEGF contribute to pathological vasculogenesis and angiogenesis [15].

### 2.2. Vascular Endothelial Growth Factor Signaling in Hemangioma

The vascular hyperpermeability associated with VEGF is also observed in IH. Of particular interest is that the increased permeability of the endothelium and the leakage of cells into the tumor interstitium contribute to the swelling and red color observed in most cutaneous lesions. Cells that are found in IH lesions include hemangioma endothelial cells (HemECs), hemangioma endothelial progenitor cells (HemEPCs), hemangioma stem cells (HemSc) and inflammatory cells [17,18,19]. Both HemEPCs and HemSc promote angiogenesis and vasculogenesis, respectively, while inflammatory cells secrete proangiogenic factors such as VEGF [20]. Thus, these inflammatory cells support neovessel formation.

An increase in VEGF promotes IH development. For instance, high levels of VEGF have been measured in the urine samples of patients with proliferating IH [1]. Also, previously, the inhibition of VEGF secretion was shown to correlate with reduced tumor cell growth in human and murine hemangioma cells [21,22]. Furthermore, targeting downstream effectors in the VEGF signaling pathway resulted in the diminished growth of endothelial cells isolated from the murine hemangiomas [23,24]. Recently, sirolimus, a drug that targets the mammalian target of rapamycin (mTOR) downstream of the VEGF pathway, was effective in regressing tumor mass in a patient with segmental IH who had been unresponsive to treatment [25].

Concerning the receptors for VEGF, research has revealed that VEGFR-2 is overexpressed in murine and human hemangioma tissue [20,26]. In contrast, VEGFR-1 expression is relatively low in hemangioma endothelial cells when compared to other endothelial cell types [8,27].

Impaired signaling through VEGFR-2 appears to play an important role in the promotion of hemangioma genesis [8]. Studies have shown that some of the patients with IH have germline mutations in VEGFR-2 or tumor endothelial marker 8 (TEM8) and that such patients appear to be at risk of developing lesions [27]. The *C482R* mutation in VEGFR-2 results in the inability of the receptor to regulate VEGFR-1 co-expression [27]. On the other hand, TEM8, which has been shown to suppress β1 integrin activity in vitro and to activate nuclear translocation of nuclear factor of activated T cells (NFAT), exhibits reduced activity [27]. Worthy of note is that the transcription of genes coding for VEGFR-1 require NFAT, and the low activity of the pathway that involves NFAT thus has a negative effect on VEGFR-1 levels [8]. Interestingly, endothelial cells isolated from hemangioma tissue show altered expression of NFAT-regulated genes [8], further supporting observations of diminished VEGFR-1 signaling in IH.

In a normal physiological setting, VEGFR-1 sequesters VEGF by binding to it and preventing it from activating VEGFR-2. In the absence of VEGFR-1’s decoy function, the VEGF levels are increased [27]. The high levels of VEGF lead to the constitutive activation of VEGFR-2 and possibly contribute to hemangioma genesis. Indeed a previous study has shown that VEGF confers increased mitogenic potential in stromal cells isolated from human hemangioma biopsies [28]. A more recent study on human IH revealed that propranolol, the first Food and Drug Administration (FDA)-approved drug for the treatment of the tumor, inhibits the growth of hemangioma endothelial cells partly by inhibiting VEGF secretion [21]. Given that these observations show that signaling through VEGF and its receptors, VEGFR-1 and VEGFR-2, is impaired in hemangioma, the pathway may be important in designing therapeutic strategies for IH.

### 2.3. Therapeutic Targeting of Vascular Endothelial Growth Factor

The first antiangiogenic drug to be approved by the Food and Drug Administration was bevacizumab, and it blocks VEGF signaling by neutralizing VEGF [13,15]. Since then, several drugs (Table 1) have been developed to target VEGF and its receptors for the treatment of tumors characterized by excessive angiogenesis [15]. The prevailing theory was that such drugs would not lead to the development of resistance since the target, namely the endothelial cell, was genetically stable [14,16]. However, in the clinic, the therapeutic benefits of these drugs have been modest and transient, and being refractory to disease remains the major drawback [16]. From investigations on the mechanism of resistance to anti-VEGF/VEGFR therapy, it appears that the stimulation of alternate proangiogenic pathways through hypoxia inducible factor-1α promotes the resumption of angiogenesis [13,15,16]. While toxicities observed with traditional chemotherapeutic drugs are not observed when VEGF neutralizing drugs are employed as monotherapy against tumor angiogenesis, the combination of these drugs with chemotherapy results in increased toxicity. Thus, the elaboration of therapies should consider, in addition to the VEGF pathway, other targets that contribute to angiogenesis, as well as combinatorial approaches that will be effective at less toxic doses.

It is also noteworthy that in several tumors, oncogenic transformations promote the angiogenic phenotype [20]. Some of these genetic alterations affect VEGF signaling, as well as other pathways that promote angiogenesis [20], thus making these genes plausible targets in the design of effective therapeutic modalities.

## 3. Oncogenic Pathways Linked to Tumor Angiogenesis

Historically, genes with the potential to induce cancer development when modified, also known as oncogenes, have been studied extensively in various neoplastic diseases [11]. However, the role of oncogenes in promoting neovascularization has recently gained considerable attention [11,12]. Oncogenic mutations have been implicated in the activation of the ‘angiogenic switch’ in tumors [12]. Some of the oncogenes that promote tumor angiogenesis are altered in IH or they influence the transcription of factors implicated in the pathogenesis of the lesion.

### 3.1. Altered Expression of Bcl-2 in Angiogenesis and Hemangioma

B-cell lymphoma-2 (*Bcl-2*) is an oncogene activated due to the t(14;18) chromosome translocation and codes for the Bcl-2 integral membrane protein, which is mainly located on the outer mitochondrial membrane [29,30]. Bcl-2 protects cells from apoptotic stimuli [29]. When overexpressed, it also delays the induction of apoptosis by various chemotherapeutic drugs [30].

**Figure 1:**
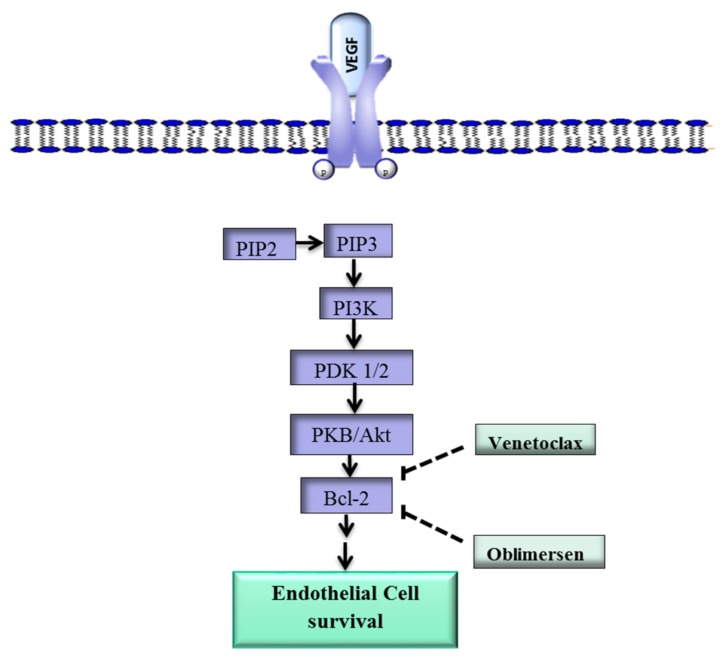
Overview of the VEGF-Bcl-2-mediated cell survival pathway. The VEGF ligand binds to VEGFR-2 and activates PI3K/Akt, which leads to the upregulation of *Bcl-2* and ultimately promotes cell survival. The drugs Venetoclax and oblimersen inhibit Bcl-2. VEGF, vegfr-2, vascular endothelial growth factor; VEGFR-2, vascular endothelial growth factor-2; Bcl-2, B-cell lymphoma 2; PKB, protein kinase B; PDK1/2, protein 3-phosphoinositide-dependent protein kinase-1 and -2; PIP2, phosphatidylinositol 4,5-bisphosphate; PIP3, phosphatidylinositol 3,4,5-trisphosphate; 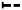 inhibit; **→**, activate.

In addition, Bcl-2 plays a role in angiogenesis via a mechanism that involves the proangiogenic factor VEGF [31]. Upon binding of VEGF to VEGFR-2, Phosphatidylinositol-4,5-bisphosphate 3-kinase (PI3k), a phospholipid that belongs to a family of enzymes that regulate a network of cellular processes, is recruited to the internal side of the cell membrane and activated through phosphorylation (Figure 1) [32]. Activated PI3k phosphorylates phosphatidylinositol 4,5-bisphosphate (PIP2) to form phosphatidylinositol 3,4,5-trisphosphate (PIP3) [33]. Phosphatidylinositol 3,4,5-trisphosphate initiates a cascade of events that lead to the phosphorylation of protein 3-phosphoinositide-dependent protein kinase-1 and -2 (PDK1/2), which in turn activate protein kinase B (PKB/Akt), a key downstream effector of PI3k [34,35]. Protein kinase B in turn upregulates Bcl-2, and the Bcl-2 protein ultimately promotes the survival of ECs [36,37].

Previous work has shown that HUVECs expressing Bcl-2 were protected from undergoing apoptosis even following the withdrawal of VEGF [36,38]. Nor et al. [38] further showed that the implantation of human microvascular endothelial cells (HDMEC) transfected with Bcl-2 into SCID mice with oral squamous cell carcinoma enhanced tumor growth. Furthermore, microvascular density increased in the mice injected with Bcl-2 transfected cells. A study by Mabeta and Pepper [26] showed that murine hemangioma overexpressed *Bcl-2* and that such expression was diminished following antiangiogenic treatment. Another study revealed that *Bcl-2* was overexpressed in proliferating IH, and that *Bcl-2* expression in involuting IH was similar to that of normal tissue [37]. These findings underscore the importance of Bcl-2 in supporting tumor angiogenesis and possibly contributing to the growth of IH. 

Various drugs have been developed to target Bcl-2. The Bcl-2 inhibitor TW37 has exhibited antiangiogenic effects in vivo in preclinical models, while S-055746 and PNT-2258 are in early stage clinical trials [39,40]. Other Bcl-2 inhibitors such as Navitoclax and Venetoclax are in phase I/II studies for several carcinomas both as monotherapy and as part of combination strategies [41,42].

Oblimersen, an antisense oligodeoxyribonucleotide that targets Bcl-2 RNA, is showing promising results in clinical studies against various cancers [43]. Oblimersen was also shown to inhibit tumor angiogenesis [43,44]. These drugs may have potential in the treatment of IH lesions that overexpress Bcl-2.

### 3.2. STAT3 Overexpression Promotes Tumor Angiogenesis

**Figure 2:**
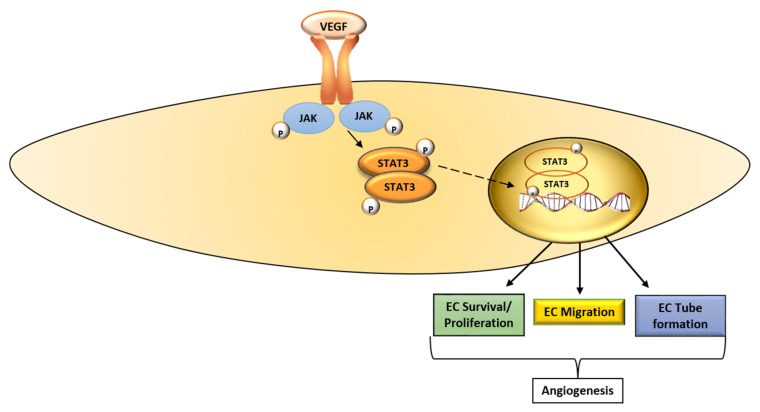
Schematic diagram of the VEGF-STAT3 signaling pathway. The binding of VEGF to VEGFR-2 activates JAK, which in turn phosphorylates STAT3. STAT3 is translocated to the nucleus where it promotes the transcription of molecules that promote endothelial cell proliferation, migration and tube formation, key EC functions that underlie angiogenesis. VEGF, vascular endothelial growth factor; JAK, Janus kinase; STAT3, signaling transducer and activator of transcription 3; EC, endothelial cell; **→**, activate; 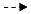, translocate into the nucleus.

The signaling transducer and activator of transcription (STAT) 3 is a member of the STAT family, and it mediates the expression of genes involved in various cellular processes [45]. Multiple pathways that transduce signals in response to cytokines and growth factors like VEGF converge on STAT3 [45,46]. The binding of VEGF to VEGFR-2 activates Janus kinases (JAK), which in turn phosphorylate STAT3 (tyr 705) [45,47]. This leads to the dimerization of STAT3 monomers through phosphotyrosine-Src homology 2 (SH2) and the translocation of the dimer to the nucleus (Figure 2). In the nucleus, the STAT3 dimer promotes the transcription of target genes involved in various stages of the angiogenic process [45]. Under normal physiological conditions, STAT3 activation is transient; however, in tumors, STAT3 is activated perpetually [45,46]. Activated STAT3 contributes to tumor progression, as well as resistance to treatment [46].

STAT3 appears to play a role in IH as phosphorylated STA3 was found to be highly expressed in human hemangioma endothelial cells [5]. STAT3 was also shown to be overexpressed in the endothelium of IH biopsies [48]. Furthermore, in a meta-analysis study of IH, STAT3 was one of the regulators identified through gene mapping [49].

Another separate study revealed that propranolol suppressed the expression of phospo-STAT3 in hemangioma cells [5], making STAT3 an attractive potential therapeutic target.

The STAT3 pathway can be disrupted at different levels [45]: (i) through the inhibition of upstream receptors that activate signals that converge on STAT3; (ii) through the prevention of dimerization by inhibiting the phosphorylation of the SH2 domain on STAT3; (iii) through the prevention of STAT3 from binding to DNA and (iv) through the downregulation of total STAT3 by inhibiting transcription. The drugs that target STAT3 at different levels are listed in Table 2.

Drugs such as ruxolitinib and fedratinib inhibit upstream receptors in the STAT3 pathway. Both ruxolitinib and fedratinib have been approved by the United States Food and Drug Administration (FDA) for the treatment of rheumatoid arthritis and are in phase I/II studies for the treatment of various cancers [45]. Several other drugs that target STAT3 are at various stages of clinical development, although some of the drugs have not progressed beyond phase I/II due to toxicity or poor efficacy [46]. Effective STAT3 inhibitors may have potential in the treatment of problematic IH.

### 3.3. Possible Role of K-Ras in Hemangioma Growth

The Kirsten rat sarcoma oncogene homologue (K-ras) encodes the GTP-ase protein K-ras, which regulates the cell cycle and, when mutated, promotes uncontrolled cell division [11]. There is strong evidence to show that K-ras mutations are also associated with alterations in vascular homeostasis [50,51]. Sustained K-ras activation in vascular endothelial cells promotes the by-passing of senescence and supports proliferation [52]. Furthermore, ‘enforced’ expression of K-ras has been shown to contribute to the increased expression of VEGF [53,54].

The proangiogenic effects of K-ras have been attributed in part to the oncogene’s negative regulation of thrombospondin, an endogenous inhibitor of angiogenesis. Studies have revealed a link between K-ras-mediated cellular transformation and the downregulation of thrombospondin-1 (TSP-1), which leads to increased angiogenesis [52]. The *K-ras* gene is mutated in several benign and malignant neoplasms. Furthermore, a number of vascular tumors carry *K-ras* mutations [52]. In the context of IH, it has been shown that TSP-1 is downregulated in tissue biopsies of proliferating IH [62]. Such downregulation of TSP-1 was not observed in involuting IH. The suppression of a negative regulator of angiogenesis, namely TSP-1, may contribute to the excessive vascularization observed in proliferating IH. In addition, given the role of K-ras in regulating TSP-1 expression and the association of overexpressed TSP-1 with growing IH, it is plausible that the targeting of *K-ras* mutations might be beneficial. Further studies are necessary to explore a possible link between mutated K-ras and IH and to determine any possible therapeutic benefit in targeting the mutated form of the gene or the protein, although the development of such drugs is still in its infancy.

## 4. Tumor Suppressors in Angiogenesis and Hemangioma Development

Tumor suppressor genes, also known as antioncogenes or oncosuppressors, are genes that encode proteins that regulate cell growth and proliferation [63]. A number of tumor suppressor genes have been shown to play a role in the promotion of tumor angiogenesis. Some of the oncosuppressors are mutated or inactivated in IH.

### 4.1. Phosphatase and Tensin Homolog Is Downregulated in Hemangioma

Phosphatase and tensin homolog deleted on chromosome 10 (PTEN) is a tumor suppressor protein encoded by the *PTEN* gene [34]. It dephosphorylates PIP3, thereby suppressing the phosphoinositol-3-kinase (PI3K)/Akt pathway [64]. Given that the PI3K/Akt pathway regulates processes such as cell survival, proliferation and migration, PTEN negatively affects these processes [64,65]. In addition, PTEN regulates angiogenesis by modulating the expression of hypoxia inducible factor 1-α (HIF1-α) [65]. Hypoxia inducible factor 1-α is involved in the tissue’s adaptive response to hypoxia [65]. Indeed, HIF1-α has been reported to be increased in specimens of IH patients [8].

Loss of PTEN promotes the expression of HIF1-α and supports angiogenesis [66], while the restoration of wild-type PTEN results in the reduced expression of HIF1-α, leading to angiogenesis inhibition [66]. Thus, PTEN loss may in part potentiate the excessive angiogenesis observed in IH. 

Indeed, PTEN loss of function due to somatic mutations has been observed in some patients with vascular anomalies and also in preclinical models of hemangioma [67]. These observations make the delivery of PTEN to HemECs an attractive therapeutic option to consider.

Although there have been studies that showed effective delivery of functional PTEN into neoplastic cells, the design of therapies that are directed at tumor suppressors poses a challenge. It is conceivable that in the future, the combination of current strategies used to treat IH with PTEN targeting approaches may be of benefit in the treatment of IH.

### 4.2. p53 Modulates Hypoxia Inducible Factor and Angiogenesis in IH

**Figure 3:**
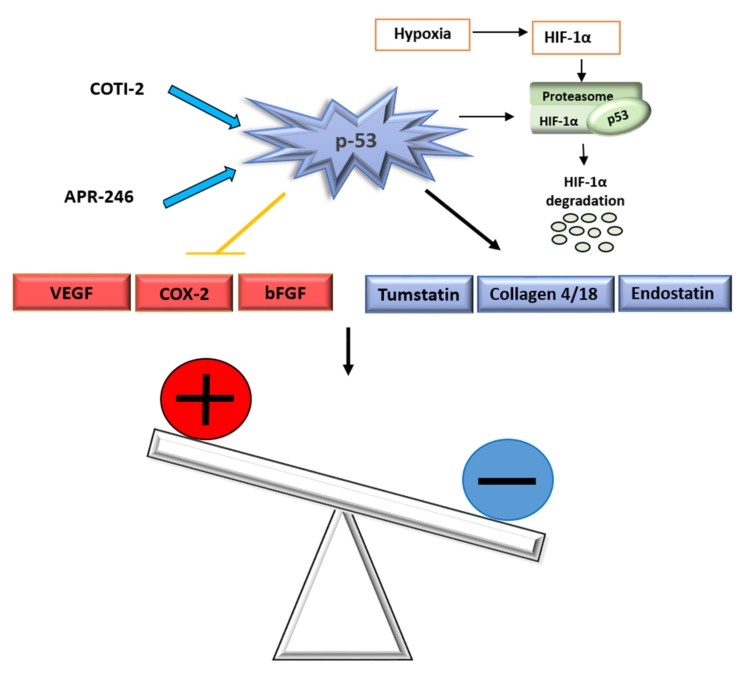
p53 negatively regulates angiogenesis. It inhibits proangiogenic proteins VEGF, COX-2 and bFGF and stimulates angiogenesis inhibitors tumstatin, endostatin and type 4 and type 8 collagen, thus tilting the scale in favor of anti-angiogenesis. HIF-1α, hypoxia inducible factor-1α VEGF, vascular endothelial growth factor; COX-2, cyclooxygenase; bFGF, basic fibroblast growth factor, −, anti-angiogenesis; +, proangiogenesis; 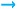, drug inhibition; 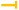, suppress; **→**, activate/stimulate.

*p53* is a tumor suppressor gene that codes for the p53 tumor suppressor protein (Tp53) [68,69]. The p53 protein is activated through phosphorylation in response to cellular stress or insult [69]. In turn, p53 can induce cell growth arrest, activate DNA repair or induce apoptosis [68,70]. p53 also suppresses the hypoxia sensing system (Figure 3) and thus plays a role in the regulation of angiogenesis [71]. Hypoxia is an important driver of angiogenesis and tumor progression. p53 inhibits angiogenesis by suppressing the hypoxia sensing system. 

The central regulatory component that responds to oxygen deprivation (hypoxia) and, as a consequence, upregulates the production of new blood vessels is called hypoxia inducible factor (HIF). Hypoxia inducible factor is a heterodimer transcription factor comprised of two subunits, HIF-1α and HIF-1β, and HIF-1α in particular is key in the cell’s response to hypoxia [71]. The biological effects of HIF-1α are derived from its ability to transcriptionally activate a variety of genes that are key to the cell’s response to oxygen deprivation, such as the gene encoding the proangiogenic factor VEGF [71,72]. Hypoxia has been postulated as a significant player in the etiology of IH [1,6,7]. Furthermore, HIF-1α is overexpressed in IH, and such expression diminishes in involuting lesion.

p53 affects the process of angiogenesis at multiple levels [70,71,72,73]. In addition to inhibiting the transcription of HIF-1A, it has a direct effect on a number of proangiogenic molecules. The binding of p53 to the transcription factor *Sp*1 inhibits VEGF expression during hypoxia by blocking Sp1’s ability to bind the VEGF promoter and to activate VEGF transcription [72]. Thus, p53 inhibits VEGF. Studies have further shown that wild-type p53 inhibits *COX-2*, ultimately suppressing angiogenesis [73]. p53 also inhibits the proangiogenic factor basic fibroblast growth factor (bFGF) through direct repression of the bFGF basal core promoter, as well as through suppressing the expression of the *bFGF-BP* gene [74]. It is interesting to note that p53 has evolved redundant mechanisms to inhibit two of the most potent secreted proangiogenic factors, namely VEGF and bFGF, and that these factors are also associated with hemangioma development. 

p53 can inhibit angiogenesis by upregulating or activating endogenous angiogenesis inhibitors [72]. Previous studies have revealed that p53 activates the α(II) collagen *prolyl-4-hydroxylase* gene [75]. The transcriptional activation of this gene leads to the release of the angiogenesis inhibitors tumstatin and endostatin (Figure 3) [75,76]. Furthermore, an association between p53 and the production of type 4 and 18 collagen fragments has been observed (Figure 3) [75]. Both of these fragments have antiangiogenic properties.

Several clinical studies have revealed a link between p53 mutations and tumor vascularization [31,77]. Human prostate cancer expressing mutated p53 is characterized by a marked increase in microvessel density (MVD) [78]. Similar correlations between p53 status and MVD were observed in colon, head and neck, as well as breast cancers [78,79]. Likewise, reduced p53 expression has been observed in proliferating IH [80]. These observations may underscore the importance of p53 in limiting angiogenesis.

There is a low expression of p53, as well as p53-related genes, including *p53 inducible gene 3* (*PIG3*) and *p53 upregulated modulator of apoptosis* (*PUMA*) in human hemangioma endothelial cells (HemECs) [80].

Further, propranolol has been shown to induce apoptosis in human hemangioma endothelial cells partly by activating p53 [81]. Another chemotherapeutic used to treat IH, bleomycin A-5, was also shown to upregulate p53 in human hemangioma cells [80]. Earlier clinical trials targeting the p53 pathway have been disappointing; however, as a result there is increasing effort to design therapeutic strategies that will yield a beneficial outcome. At present COTI-2, a small molecule which restores mutant, APR-246 an activator of mutant p53, are in clinical trials for various malignancies [79]. It is likely that these approaches may be of benefit to IH.

### 4.3. Deregulation of Kiss1 Metastasis-Suppressor Is Associated with IH

In a previous study, *Kiss1* metastasis-suppressor (Kiss1) was underexpressed in endothelial cells isolated from human hemangioma tissue [82]. The *Kiss1* gene is a tumor suppressor that codes for a protein that regulates cancer cell metastasis by suppressing matrix metalloproteinase 9 (MMP9) [83]. The gene also suppresses angiogenesis. Concerning IH, Stiles et al. [82] further showed that the low expression of *Kiss1* associated with haemangioma endothelial cells, was not observed in other endothelial cell types. Perhaps *Kiss1* may serve as a predictive biomarker to monitor IH response to therapy. Further studies are required to determine its expression in the various phases of the IH life cycle. 

### 4.4. Cyclin-Dependent Kinase Inhibitor 2A

Cyclin-dependent kinase inhibitor 2A (CDKN2A) is a gene that codes for two proteins, the INK4 family member p16 (or p16INK4a) and p14arf [84]. These proteins regulate the cell cycle and function as tumor suppressors. The gene was shown to be downregulated in hemangioma cells. These observations correlated with alterations in the cell cycle of hemangioma cells when compared to normal dermal microvascular endothelial cells [82]. Similar to kiss1, the expression of CDKN2A in the various phases of IH requires investigation in order to determine a possible link with disease progression. However, given the selective expression of these genes in IH compared to normal endothelial cells, they may be useful as predictive biomarkers to determine the effectiveness of therapeutic approaches employed in IH, those modalities that are in the developmental phase. 

## 5. Conclusions

There is strong evidence to show that the acquisition of mutations in proto-oncogenes contributes to the onset of the ‘angiogenic switch’, especially by enhancing VEGF signaling. In addition, loss of function due to genetic lesions in oncosuppressors promotes angiogenesis. Vascular endothelial growth factor signaling is a requirement for vasculogenesis and is also key during the process of angiogenesis. These two process underlie the development of hemangiomas in juveniles. Some of the oncogenes and oncosuppressors that are associated with defective VEGF signaling are altered also in IH, while some genes regulate factors linked to the neoplasm. As such, an in-depth understanding of the roles of oncogenes and oncosuppressors in hemangioma genesis may provide the basis for therapeutic targeting. Furthermore, understanding the profile of the lesions in each context is necessary and may lead to a more sustainable approach as treatment can be adapted depending on the genetic profile of the tumors. Indeed, some of the drugs in this review may have future application in IH either as monotherapies or as part of combination therapeutic strategies.

## Figures and Tables

**Table 1:** Drugs targeting the vascular endothelial growth factor family of ligands and their receptors.

Drug	Target	Disease Indication	Reference
Aflibercept	VEGF-A, VEGF-B, PlGF	Broad spectrum of malignancies	[13,14]
Axitinib	VEGFR-1, VEGFR-2, VEGFR-3	Renal cell carcinoma	[13]
Bevacizumab	VEGF-A	Breast, ovarian, metastatic colorectal and non-small cell lung cancers, recurrent glioblastoma	[13,15]
Lenvatinib	VEGF-A	Thyroid cancer	[16]
Pegaptanib	VEGF-A	Age-related macular degeneration	[14]
Pazopanib	VEGFR1, VEGFR2, VEGFR3	Advanced soft tissue sarcoma	[13]
Sorafenib	VEGFR1, VEGFR2, VEGFR3	Renal cell cancer, hepatocellular carcinoma	[13,14]
Sunitinib	VEGFR1, VEGFR2, VEGFR3	Renal cell carcinoma, pancreatic neuroendocrine	[16]
Regorafenib		Refractory metastatic colorectal cancer	[14]
Ramucirumab	VEGFR-2	Metastatic colorectal, gastric/gastro-esophageal, non-small-cell lung cancer	[14]
Vatalanib	VEGFR1, VEGFR2, VEGFR3	Solid tumors	[14]

**Table 2:** Oncogenes linked to IH and the drugs that inhibit these genes.

Target	Drug	Phase of Development	Reference
**Bcl-2**			
	S-055746	I	[55]
	PNT-2258	II	[41]
	Navitoclax	I/II and III	[56]
	Venetoclax	I/II	[41,42]
	Oblimersen	I/II and III	[43,44]
**STAT3**			
Upstream tyrosine kinase inhibitors	Ruxolitinib	I–III	[45]
	Dasatinib	III	[57]
	Fedratinib	I/II and III	[58]
	Tofacitinib	I/II	[45]
SH2 domain inhibitors	OPB-31121	I/II	[46]
	OBP-51602	I/II	[59]
STAT3 DNA-binding domain inhibitors	CPA-1	Preclinical	[46]
	Cyclic STAT3 decoy	Preclinical	[45]
STAT3 transcription inhibitors	AZD9150	I/II	[60]
	STAT3 decoy oligonucleotide	0	[61]

## Abbreviations

Bcl-2	B-cell lymphoma 2
bFGF	Basic fibroblast growth factor
COX-2	Cyclooxygenase 2
EC	Endothelial cell
GTP-ase	Guanosine triphosphatase
HemECs	Hemangioma endothelial cells
HemEPCs	Hemangioma endothelial progenitor cells
HemSc	Hemangioma stem cells
HIF-1α	Hypoxia inducible factor-1α
IH	Infantile hemangioma
JAK	Janus kinases
K-ras	Kirsten rat sarcoma oncogene homologue
mTOR	Mammalian target of rapamycin
NFAT	Nuclear translocation of nuclear factor of activated T cells
PDK1/2	Protein 3-phosphoinositide-dependent protein kinase-1 and -2
PI3k	Phosphatidylinositide 3-kinases
PKB	Protein kinase B
PIP2	Phosphatidylinositol 4,5-bisphosphate
PIP3	Phosphatidylinositol 3,4,5-trisphosphate
PTEN	Phosphatase and tensin homolog deleted on chromosome 1
TSP-1	Thrombospondin-1
STAT3	Signaling transducer and activator of transcription
VEGF	Vascular endothelial growth factor
VEGFR-1	Vascular endothelial growth factor receptor-1
VEGFR-2	Vascular endothelial growth factor receptor-2
